# Benchmarking large-language-model vision capabilities in oral and maxillofacial anatomy: A cross-sectional study

**DOI:** 10.1371/journal.pone.0335775

**Published:** 2025-10-28

**Authors:** Viet Anh Nguyen, Thi Quynh Trang Vuong, Van Hung Nguyen

**Affiliations:** 1 Faculty of Dentistry, Phenikaa University, Hanoi, Vietnam; 2 Private Practice, Viet Anh Orthodontic Clinic, Hanoi, Vietnam; 3 Faculty of Medicine, Vinh Medical University, Nghe An, Vietnam; Peking University, CHINA

## Abstract

**Background:**

Multimodal large-language models (LLMs) have recently gained the ability to interpret images. However, their accuracy on anatomy tasks remains unclear.

**Methods:**

A cross-sectional, atlas-based benchmark study was conducted in which six publicly accessible chat endpoints, including paired “deep-reasoning” and “low-latency” modes from OpenAI, Microsoft Copilot, and Google Gemini, identified 260 numbered landmarks on 26 high-resolution plates from a classical anatomic atlas. Each image was processed twice per model. Two blinded anatomy lecturers scored responses, including accuracy, run-to-run consistency, and per-label latency, which were compared with χ² and Kruskal–Wallis tests.

**Results:**

Overall accuracy differed significantly among models (χ² = 73.2, *P* < 0.001). OpenAI o3 achieved the highest correctness (53.1%), outperforming its sibling GPT-4o and both Copilot variants, but required the longest inference time. Musculoskeletal structures were recognised more accurately than neurovascular targets, reflecting the greater visual complexity of fine vessels and nerves. Consistency ranged from 43.5% (Gemini Flash) to 65.0% (GPT-4o); deeper modes improved stability for Copilot and Gemini but not accuracy. Median per-label latency spanned three orders of magnitude, from 0.5 s for Gemini Flash to 33 s for o3.

**Conclusions:**

Currently, publicly available multimodal LLMs can only moderately identify oral and maxillofacial landmarks, and no endpoint is sufficiently reliable to serve as a stand-alone answer key. Higher accuracy was achievable with a trade-off in latency, highlighting the need for domain-specific tuning and human oversight. This atlas benchmark study introduced here provides a reproducible yardstick for future model refinement and educational integration.

## 1. Introduction

Large-language models (LLMs) have progressed rapidly from text-only transformers to multimodal engines capable of parsing images, audio, and code in real time [1]. These systems already equal or surpass average human examinees on several health-licensing examinations when questions are presented as text, and educators have begun to embed chatbots into flipped-classroom and “just-in-time” learning workflows [2–4]. Dentistry has mirrored this trajectory, with GPT-4 class models routinely scoring above the pass mark on National Board Dental Examination items and specialty in-service tests, while students report using ChatGPT for rapid revision and concept clarification [5–7].

Yet most published evaluations probe only the linguistic channel. In contrast, vision-language capabilities accept images alongside text and generate text grounded in the visual content. Anatomical and clinical teaching in oral and maxillofacial curricula is intrinsically visual, relying on atlases, radiographs, and operative photographs that encode spatial relations impossible to convey in prose alone. To date, peer-reviewed benchmarks of multimodal LLMs on image-dependent dental tasks are scarce, limited to small sets of radiographs or mixed text–image items with substantial verbal scaffolding [8,9]. When image-only dependence is high, accuracy can be modest [7]. Recent multimodal benchmarks in medicine show that domain-tuned vision-language models can reach strong scores on medical visual question answering datasets, but fine-grained, spatially dependent identification remains challenging [10,11]. Region-of-interest prompting and grounding notably improve performance, underscoring the importance of explicit localization for numbered anatomy tasks. No study has systematically compared the vision performance of competing commercial endpoints across the full breadth of oral and maxillofacial anatomy or in other anatomical regions. This gap obscures the true educational value and reliability of the “vision” capability now marketed by major vendors.

Accordingly, a cross-sectional, atlas-based benchmark was developed to compare six chat-based endpoints from three leading vendors, contrasting deep-reasoning and low-latency configurations in an oral and maxillofacial anatomy identification task. The rationale for contrasting these two configurations is that they represent the common trade-off between accuracy and speed in practical dental artificial intelligence (AI) usage, where both thorough reasoning and rapid responses are valued in different contexts. These vendors were selected because they represent the latest production-grade offerings publicly available from the three vendors commanding the largest market share in health-science generative-AI research, including dentistry, at the time of study, and have been cited in recent dental-education or medical-AI literature, ensuring relevance to our target domain of oral and maxillofacial anatomy [8].

Our primary hypothesis was that depth-reasoning variants would outperform their low-latency counterparts on accuracy, particularly for larger musculoskeletal landmarks where full-context reasoning offers a clearer advantage. We further posited that any accuracy gain would be offset by longer response times and, potentially, reduced run-to-run consistency. By systematically quantifying accuracy, consistency, and latency across six state-of-the-art endpoints, our study provides a head-to-head benchmark not only for oral- and maxillofacial-specific anatomical identification but also for image-based question-answering tasks in dental education more broadly.

## 2. Methods

This cross-sectional observational study was designed and reported in accordance with the Strengthening the Reporting of Observational Studies in Epidemiology (STROBE) guidelines. As this study exclusively utilized de-identified, published anatomical illustrations from an anatomic atlas and did not involve human participants or personal data, ethical approval was not required. The Institutional Review Board of Phenikaa University issued an exemption determination (no. 2025−55 dated May 12^^th^^ 2025). An a priori sample size calculation was performed using G*Power 3.1 (Heinrich–Heine–Universität Düsseldorf, Düsseldorf, Germany). Assuming a small effect size of w = 0.104, a two‐sided α of 0.05, and 90% power for a χ² test of independence with 5 degrees of freedom, the analysis indicated that at least 254 question-label pairs would be required, based on estimates reported by Nguyen et al. [8]. Accordingly, this study employed a fixed set of 260 labeled items, which were answered by each LLM.

We benchmarked six publicly accessible chat-based LLM endpoints from three leading vendors, including OpenAI (San Francisco, CA, USA), Microsoft Copilot (Redmond, WA, USA), and Google Gemini (Mountain View, CA, USA) [8,12]. For each vendor, we deliberately paired one model that prioritises depth of reasoning at the expense of latency with another that prioritises speed (low-latency mode) (Table 1). Models were accessed via their official web or API front-ends using the default temperature and safety settings current on the date of testing. Additionally, each vendor provides both “fast” and “deep-reasoning” options, allowing a controlled comparison of accuracy versus latency trade-offs.

**Table 1:** Chat-based large-language models evaluated in this study.

Vendor	OpenAI	Microsoft Copilot	Google Gemini
Deep-reasoning model	o3: flagship reasoning model released April 2025, designed to spend more time thinking on complex tasks [15]	Copilot Deep (Think Deeper): Copilot conversation mode powered by Azure OpenAI o3-mini model that enables deeper logical analysis with longer response times	Gemini Pro 2.5: high-fidelity model tuned for accuracy and complex reasoning, albeit with longer processing times
Low-latency model	GPT-4o (omni): multimodal, 2 × faster and 50% more cost-effective than GPT-4 Turbo	Copilot Quick: default Copilot mode optimised for rapid, lightweight answers (sub-second streaming in most cases)	Gemini Flash 2.5: model variant explicitly optimised for real-time usage and low latency, trading some accuracy for speed

Twenty-six high-resolution plates were extracted directly from the digital 8th edition of Netter’s Atlas of Human Anatomy, chosen for its authoritative status and widespread use in anatomical education and clinical practice [13,14]. Each plate was annotated with ten predefined, consecutively numbered landmarks (labels 1–10), each rendered as a white circular marker containing the number and placed directly on its corresponding anatomical structure. No arrows or leader lines were employed, as preliminary testing showed that arrow- or line-based labels led to misidentification of the structure nearest the label rather than the true target at the arrowhead and yielded lower accuracy [15,16]. To minimize marker-structure overlap, we preferentially selected landmarks with sufficient surface area to accommodate the circular marker with a small safety margin. This pragmatic constraint resulted in a modest over-representation of musculoskeletal targets relative to slender neurovascular structures. Difficulty level was not used as a selection criterion because marker diameter primarily determined item visibility.

From 1^^st^^ to 10^^th^^ June 2025, each image with its embedded labels was sequentially submitted to the six LLMs using the following standardized prompt:

“You are a dental student currently taking your final examination in the oral and maxillofacial anatomy module. The exam image provided contains numbered labels that represent the components of [figure caption]. Your task is to correctly identify each anatomical structure corresponding to each numbered label. Please present your answer in a two-column table with the headers “label” and “anatomical name,” and include no additional text or notes.”

All illustrations were obtained as original digital exports rather than scanned images, preserving their native resolution. Each prompt–image pair was submitted in an isolated session. To prevent context carryover and bias, each model interaction was programmatically terminated immediately after the response was returned. The full chat history, including the prompt and associated image, was deleted from the session context, ensuring no conversational history influenced subsequent queries. Each image was processed twice by each LLM to assess response stability and consistency across repeated interactions. Response latency per label was calculated by measuring the time from prompt submission to the end of the model’s internal processing, right before any answer tokens were streamed, for each plate, and then dividing that duration by ten, corresponding to the ten labels in the prompt, thereby reducing sensitivity to network speed.

**Fig 1:**
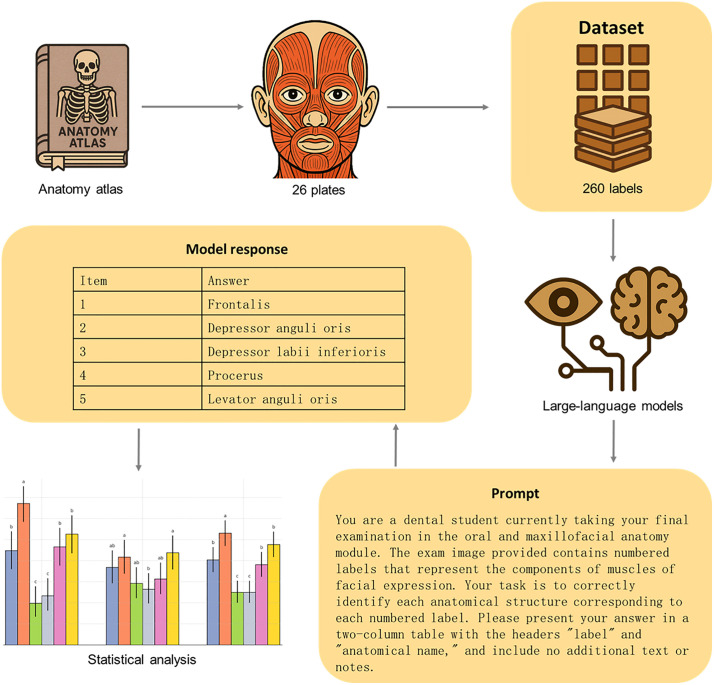
Study flowchart. The anatomy icon was drawn de novo by the corresponding author in Adobe Illustrator 2024 (Adobe Inc., San Jose, CA, USA); no external images, stock assets, or anatomical atlases were used, retrieved, traced, or adapted.

Two independent anatomy lecturers systematically evaluated each response against the ground truth derived from the authoritative anatomical sources. Both accuracy and consistency were scored as per-item binary outcomes. Accuracy was determined by whether the AI-generated responses precisely matched expert anatomical nomenclature, while consistency was assessed based on variations between first and second interactions (Fig 1). To ensure both accuracy and consistency, responses were considered correct if each anatomical structure was identified by its proper name or by an acceptable synonym such as “levator nasolabialis” or “levator labii superioris alaeque nasi”.

To quantify inter-rater reliability, both lecturers independently scored responses, with Cohen’s kappa coefficient calculated to ensure evaluator agreement. Labels were stratified into two categories, including musculoskeletal and neurovascular, and accuracy across the six groups was compared using chi-square tests, with post hoc Benjamini-Hochberg adjustments applied to the total sample as well as within each category. Consistency was similarly evaluated via chi-square tests and corresponding post hoc analyses. Given that response latency per label did not follow a normal distribution, group comparisons were conducted using the Kruskal–Wallis test, with subsequent pairwise post hoc comparisons performed using the Mann–Whitney U test adjusted with the Bonferroni correction. Statistical significance was set at α = 0.05 for all tests. Additionally, a post-hoc qualitative error review was performed by the authors during analysis to identify item types that were most prone to recurrent failures.

## 3. Results

**Fig 2:**
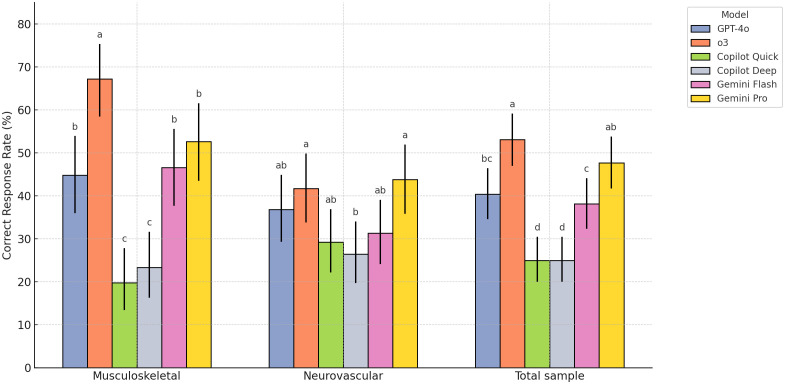
Correct-response rates and 95% confidence intervals of six large-language models when answering multiple-choice items. Superscript letters above each bar denote pairwise comparisons, with values sharing at least one common letter (a-d) do not differ significantly according to χ² tests with Benjamini-Hochberg adjustment.

Inter-rater reliability was perfect, with a Cohen’s kappa coefficient of 1, indicating complete agreement between the two lecturers. Accuracy varied markedly across models (Table 2, Fig 2). Overall, the proportion of correctly identified labels differed significantly between the six LLMs (χ² = 73.2, *P* < 0.001). o3 yielded the highest accuracy (53.1%, 95% CI 47.0–59.1), but it did not differ from Gemini Pro (P = 0.253, Benjamini-Hochberg adjusted). Copilot Quick and Copilot Deep were the lowest (each 25.0%, 20.0–30.5) and were significantly below the other models (P < 0.01). Among the remaining comparisons, Gemini Pro exceeded Gemini Flash (P = 0.036), whereas GPT-4o did not differ from Gemini Pro or Gemini Flash (P > 0.05). Performance patterns varied within anatomical sub-groups. For musculoskeletal structures (n = 116), accuracies again differed by model (χ² = 76.9, *P* < 0.001), with o3 outperforming all comparators (67.2%, 58.4–75.3) and both Copilot variants remaining least accurate (≤23.3%). In the neurovascular set (n = 144), the inter-model gap narrowed yet remained significant (χ² = 15.6, *P* = 0.008), with o3 (41.7%, 33.8–49.8) and Gemini Pro (43.8%, 35.8–51.9) sharing the top rank, while Copilot Deep trailed (26.4%, 19.7–34.0).

**Table 2:** Accuracy (%) of six large-language models in identifying anatomical labels.

Category	Musculoskeletal (n = 116)	Neurovascular (n = 144)	Total sample (n = 260)
GPT-4o	44.8% (36.0–53.9)^b^	36.8% (29.3–44.9)^ab^	40.4% (34.6–46.4)^bc^
o3	67.2% (58.4–75.3)^a^	41.7% (33.8–49.8)^a^	53.1% (47.0–59.1)^a^
Copilot Quick	19.8% (13.4–27.8)^c^	29.2% (22.2–36.9)^ab^	25.0% (20.0–30.5)^d^
Copilot Deep	23.3% (16.3–31.6)^c^	26.4% (19.7–34.0)^b^	25.0% (20.0–30.5)^d^
Gemini Flash	46.6% (37.7–55.6)^b^	31.3% (24.1–39.1)^ab^	38.1% (32.3–44.1)^c^
Gemini Pro	52.6% (43.5–61.5)^b^	43.8% (35.8–51.9)^a^	47.7% (41.7–53.8)^ab^
P value	< 0.001	0.008	< 0.001

Within each column, values sharing at least one common superscript letter (a-d) are not significantly different from one another (pairwise χ² tests of column proportions with Benjamini-Hochberg adjustment, α = 0.05). Values with no letter in common differ significantly.

Consistency differed significantly among the six LLMs (χ² = 54.8, *P* < 0.001, Table 3). For the total sample, GPT-4o (65.0%), Copilot Deep (63.5%), and Gemini Pro (61.9%) showed the highest overall consistency, with no significant differences between them after Benjamini-Hochberg adjustment. o3 (56.9%) overlapped with both the top group and Copilot Quick (51.2%), while Gemini Flash (43.5%) was significantly lower than most others. Patterns within anatomical sub-groups were similar but not identical. For musculoskeletal structures, consistency again varied by model (χ² = 28.7, *P* < 0.001). Gemini Pro achieved the highest numerical value (73.3%) but did not differ significantly from GPT-4o, o3 (both 68.1%), or Copilot Deep (60.3%). Copilot Quick was lower (42.2%) and differed from the higher-performing group, whereas Gemini Flash (56.9%) did not differ from Copilot Quick. In the neurovascular set, inter-model differences persisted (χ² = 26.1, *P* < 0.001). Copilot Deep (66.0%) and GPT-4o (62.5%) led without a significant difference between them. Gemini Pro (52.8%) and o3 (47.9%) were lower, Copilot Quick (58.3%) overlapped with both tiers, and Gemini Flash (32.6%) was significantly below all other models.

**Table 3:** Consistency (%) of six large-language models across two repeated interactions.

Category	Musculoskeletal(n = 116)	Neurovascular(n = 144)	Total sample(n = 260)
GPT-4o	68.1% (59.3–76.1)^ab^	62.5% (54.4–70.1)^ab^	65.0% (59.1–70.6)^a^
o3	68.1% (59.3–76.1)^ab^	47.9% (39.9–56.0)^c^	56.9% (50.9–62.8)^ab^
Copilot Quick	42.2% (33.5–51.3)^c^	58.3% (50.2–66.2)^abc^	51.2% (45.1–57.2)^bc^
Copilot Deep	60.3% (51.3–68.9)^ab^	66.0% (58.0–73.3)^a^	63.5% (57.5–69.1)^a^
Gemini Flash	56.9% (47.8–65.6)^bc^	32.6% (25.4–40.6)^d^	43.5% (37.5–49.5)^c^
Gemini Pro	73.3% (64.7–80.7)^a^	52.8% (44.6–60.8)^bc^	61.9% (55.9–67.7)^a^
P value	< 0.001	< 0.001	< 0.001

Within each column, values sharing at least one common superscript letter (a-d) are not significantly different from one another (pair-wise χ² tests of column proportions with Benjamini-Hochberg adjustment, α = 0.05). Values that do not share a letter differ significantly.

**Fig 3:**
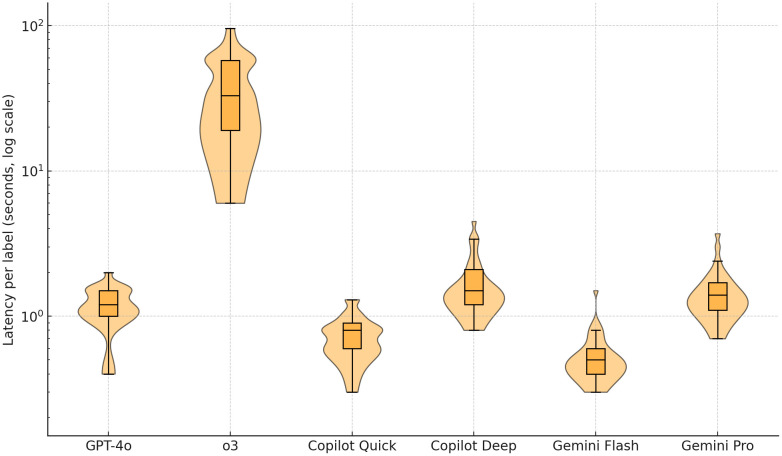
Log-scale violin-and-box plots showing the distribution of per-label response latency (seconds) for six large-language models.

Median per-label response time differed markedly among models (Kruskal–Wallis H = 1190.8, P < 0.001, Table 4, Fig 3). Across the total sample, Gemini Flash was the fastest (0.5 s, interquartile range 0.2 s), followed by Copilot Quick (0.8 s, 0.3 s), GPT-4o (1.2 s, 0.5 s), Gemini Pro (1.4 s, 0.6 s), Copilot Deep (1.5 s, 0.9 s), and o3 (33.0 s, 38.7 s; all pair-wise P < 0.001, Bonferroni adjusted). The extremes were consistent across anatomical subgroups, with Gemini Flash remaining the fastest and o3 the slowest, while the middle ranks shifted modestly between sets. For musculoskeletal labels (H = 521.9, P < 0.001), Gemini Flash (0.5 s) remained the quickest, while o3 required a median of 53.1 s. For neurovascular labels (H = 680.5, P < 0.001), Gemini Flash (0.5 s) was again fastest, followed by Copilot Quick (0.8 s), GPT-4o (1.1 s), Gemini Pro, Copilot Deep (both 1.5 s), and o3 remained the slowest at 32.2 s.

**Table 4:** Per-label response latency (median seconds, interquartile range) of six large-language models.

Category	Musculoskeletal(n = 116)	Neurovascular(n = 144)	Total sample(n = 260)
GPT-4o	1.3 (0.3)^c^	1.1 (0.5)^c^	1.2 (0.5)^c^
o3	53.1 (38.7)^e^	32.2 (38.8)^e^	33.0 (38.7)^f^
Copilot Quick	0.8 (0.5)^b^	0.8 (0.2)^b^	0.8 (0.3)^b^
Copilot Deep	1.5 (1.8)^d^	1.5 (0.3)^d^	1.5 (0.9)^e^
Gemini Flash	0.5 (0.1)^a^	0.5 (0.2)^a^	0.5 (0.2)^a^
Gemini Pro	1.2 (0.4)^c^	1.5 (0.8)^d^	1.4 (0.6)^d^
P value*	< 0.001	< 0.001	< 0.001

Kruskal–Wallis tests of independent samples comparing median latency across the six models. Superscript letters indicate groups that do not differ significantly within each column (pair-wise Mann–Whitney U tests with Bonferroni correction). Values that share no letter are significantly different.

**Fig 4:**
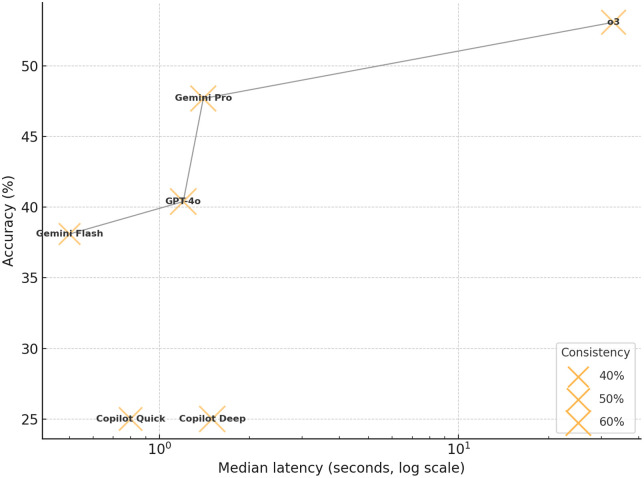
Accuracy-versus-latency trade-off for six large-language models. Each cross marker locates a model by its median response latency (seconds, log-scale) and overall accuracy. Marker size is proportional to consistency across repeated interactions.

We pooled accuracy, latency, and consistency into a Pareto plot (Fig 4) to illustrate the overall trade-off among the three metrics. This visualization highlights the models that lie on the performance frontier. Across the whole sample, depth-reasoning endpoints were significantly more accurate for OpenAI (Δ = 12.7%, 95% CI 4.1–21.0; P = 0.006, Benjamini-Hochberg adjusted) and Google Gemini (Δ = 9.6%, 95% CI 1.1–17.9; P = 0.036), but not for Microsoft Copilot (P = 1). Latency consistently favored the speed-optimized endpoints, with Hodges–Lehmann estimates of the median differences being 32.3 s (95% CI, 31.3–38.0) for o3 versus GPT-4o, 0.7 s (95% CI, 0.7–0.8) for Copilot Deep versus Copilot Quick, and 0.8 s (95% CI, 0.8–0.9) for Gemini Pro versus Gemini Flash.

**Fig 5:**
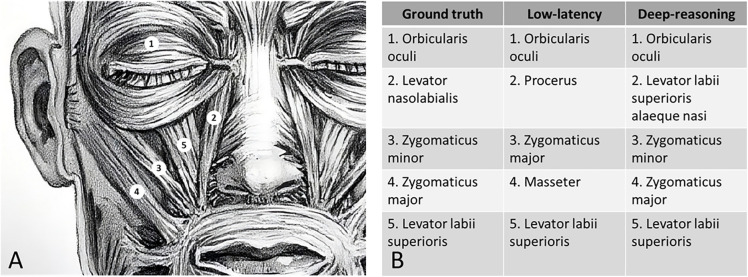
Example of landmark-name identification on an anterior facial muscle plate. **(A)** Facial musculature redrawn for illustrative purposes. **(B)** Ground-truth terms with the corresponding outputs.

To illustrate typical successes and confusions, Fig 5 shows a redrawn facial muscle plate with numbered landmarks alongside ground-truth terms and the outputs from the low-latency and deep-reasoning endpoints.

## 4. Discussion

This cross-sectional, atlas-based evaluation reveals that the vision capabilities of publicly accessible LLMs remain heterogeneous and are strongly modulated by each vendor’s accuracy-latency trade-off. Consistent with our a priori expectation that depth-reasoning engines would outperform low-latency variants, OpenAI’s o3 delivered the highest overall accuracy, particularly for musculoskeletal landmarks. Yet the superiority of o3 narrowed, or even vanished, within neurovascular structures, where Gemini Pro matched its performance while retaining far shorter response times. These findings, therefore, only partly validate our central hypothesis, underscoring that domain-specific image complexity, rather than a model’s nominal “deep-reasoning” designation alone, determines diagnostic yield.

Accuracy gains emerged only when the vendor’s “deep-reasoning” tier was backed by genuinely broader model capacity. OpenAI’s o3, powered by a larger architecture than GPT-4o, delivered a clear accuracy boost, albeit at the cost of markedly slower responses and a slight dip in run-to-run stability. Microsoft’s Copilot Deep, by contrast, added decoding time without materially improving correctness. Its sole benefit was a modest increase in answer consistency, evidence that the two Copilot modes likely share the same compact backbone and differ mainly in sampling depth. Google’s Gemini Pro occupied a middle ground, with accuracy edging upward relative to Gemini Flash but not enough to reach statistical significance, while consistency improved and latency remained within a “classroom-friendly” window. Collectively, these patterns suggest that longer inference alone cannot compensate for limited model capacity. Only when deeper sampling is coupled with a more capable network, as in o3, does it translate into meaningfully better answers. Educators, therefore, face a nuanced trade-off in which OpenAI provides the clearest lever for higher accuracy, whereas Copilot and Gemini deep modes may be preferable when steadier, though not necessarily more correct, responses are desired over raw speed.

A plausible mechanism for the divergent performance profiles lies in the geometric complexity and relative size of the target structures. Muscles, ligaments, and bones occupy broad, high-contrast regions; when a circular label is placed, it generally falls entirely within the intended structure, allowing the vision encoder in o3 to sample sufficient spatial context and minimise confusion with neighbouring anatomy. By contrast, vessels and nerves are slender, tortuous, and frequently partially obscured by overlying tissue, so a label may cover only a small segment of the structure while masking adjacent, visually similar background. The mixed signal captured in such patches demands more precise spatial reasoning, eroding the architectural advantage that o3 displayed on the musculoskeletal set and narrowing the accuracy gap among models in the neurovascular subset.

Across all six multimodal LLM endpoints, we observed a systematic failure to recognise a cluster of deep structures, such as the deep portion of the masseter, the anterior belly of the digastric, and the body of the sphenoid bone. These labels are characteristically small or thin, reside in overlapping tissue planes, carry hierarchical nomenclature that embeds a parent-subunit relationship, and are sparsely represented in mainstream biomedical corpora. Such features diminish their visual salience in two-dimensional atlases, destabilise text-image embeddings, and expose current limitations in the models’ three-dimensional spatial reasoning and fine-grained anatomical parsing. Remediating this blind spot will require training datasets enriched with volumetric head-and-neck imaging and ontology-linked, segment-level annotations, coupled with curriculum strategies that emphasise low-frequency, high-granularity anatomy.

In comparison with prior LLM work in dental education, text-based assessments dominate the published literature and show a clear, generation-dependent performance gradient. At the frontier, domain-aligned models have reached expert-level performance on medical question answering [17]. In a board-style multiple-choice test drawn from U.S. licensing resources, GPT-4 answered 76.9% correctly versus 61.3% for GPT-3.5, a statistically significant 15-point gain [18]. In free-text clinicopathologic-conference style diagnostics, ChatGPT-4o also performed strongly, correctly identifying 78% maxillofacial cases [19]. A similar gap appeared on the 2023 Periodontic In-Service Examination, where GPT-4 reached 73.6% while its predecessor managed 57.9% [3]. Licensing-level datasets in other jurisdictions confirm the trend, with GPT-4 achieving roughly 75% accuracy on the Korean National Dental Hygienist Examination, again outperforming Gemini, Bing/Copilot, and GPT-3.5 across both English and Korean versions [20]. Specialty-focused MCQs can push the ceiling even higher; on a corpus of 123 oral-radiology questions from Türkiye’s Dental Specialty Admission Exam, GPT-4o scored 86.1%, eclipsing Bard (61.8%), GPT-3.5 (43.9%), and Copilot (41.5%) [5]. Collectively, these studies place modern GPT-4-class chatbots in the 70–85% range for text-only dental knowledge checks, well above older models yet still well short of perfect recall.

Evidence for free-response or open-ended tasks is thinner but broadly consonant with the MCQ pattern. In a final-year written periodontology examination comprising 20 short-answer questions, GPT-4 produced mean scores of 78% (first run) and 77% (third run), significantly exceeding the student cohort’s 60% average, although performance varied between attempts [21]. When three chatbots answered 31 frequently-asked patient questions about dental prostheses, Gemini delivered the highest quality (mean 4.6/5) while ChatGPT’s responses were more verbose and written at a higher reading level, raising readability concerns despite overall factual adequacy [6]. Such findings underline that, even when accuracy is acceptable, consistency and audience-appropriate language remain variable and warrant expert oversight.

Robust, peer-reviewed benchmarks that probe LLMs with educational image-based dental questions remain sparse. Beyond our atlas experiment, we located only two English-language studies, and even they contained comparatively few image items (47 and 160, respectively) relative to the thousands of text questions used elsewhere. Nguyen et al. embedded only 47 image-based MCQs, each accompanied by diagrams, radiographs, or clinical photographs, within a 1490-item United States National Board Dental Examination corpus. The three multimodal chatbots that could ingest pictures (ChatGPT-4o, Copilot, Claude 3.5) answered these items with 61.7–63.8% accuracy, whereas Gemini achieved 31.9%, and two text-only models (Mistral, Llama) either performed markedly worse or skipped the visuals entirely [8]. Although this accuracy is modestly higher than in our atlas benchmark, most of Nguyen’s questions provided substantial textual context and used the image merely as a supplementary cue, which likely inflated scores relative to tasks like ours, where the correct answer is exclusively image dependent.

Tasks that require selecting the correct structure among multiple numbered candidates resemble grounded visual question answering. Region-of-interest methods outperform generic prompting on such localized queries, suggesting that explicit spatial cues are pivotal for anatomy-label identification [22,23]. Morishita et al. evaluated 160 image-rich questions from the 2023 Japanese National Dental Examination and found a markedly lower overall accuracy of 35.0% for GPT-4 Vision, with performance deteriorating further whenever a single item contained multiple or highly detailed images [9].

Findings in radiology mirror this pattern. GPT-4V performs well on text-only board-style items but drops on image-dependent questions, with accuracy only partially recovering under optimized sampling temperatures, and radiologists generally still retain an edge [24,25]. A recent meta-analysis of visual-language models in medical imaging aligns with these trends [26]. Multi-image reasoning further depresses scores, highlighting the burden of visual complexity and spatial disambiguation relative to mixed text-image items [27–30]. Thus, our findings sit squarely between these two extremes, reinforcing the notion that LLM accuracy declines as visual dependency and image complexity increase, and that mixed text-image items may overestimate a model’s true “vision” capability. Cross-specialty evaluations report variable but improving performance of frontier chatbots across imaging subspecialties, while early dental-radiology studies with GPT-4V on periapicals indicate feasibility yet emphasize the need for domain tuning and careful validation [31–33]. Beyond benchmarking, early clinical integrations of AI illustrate near-term utility for dental digital workflows [34].

Persistent errors on small, deep, and plane-overlapping structures point to a gap in supervision and data rather than a limit of model capacity. A focused adaptation path is to train on standardized segment-level labels paired with volumetric head-and-neck imaging or three-dimensional encodings, apply parameter-efficient fine-tuning such as instruction tuning or low rank adaptation or adapter layers on paired image and label sentences with explicit hierarchical synonyms, and constrain decoding through retrieval from a controlled taxonomy [35]. A curriculum that upweights rare, highly granular labels and makes hierarchy relations explicit, together with uncertainty calibration on rare classes, is likely to yield the largest gains.

From an educational standpoint, our data highlight the tension between utility and reliability when deploying LLMs in anatomy teaching. OpenAI o3 ranks clearly above the other endpoints for accuracy, yet it still misidentifies roughly one label in every two. Students, therefore, cannot treat any current model as a definitive answer key but rather as an adjunct that augments, not replaces, expert feedback. The sub-second response times delivered by GPT-4o and Gemini Flash are attractive for “just-in-time” look-ups during dissection or flipped-classroom sessions, whereas the deeper, slower variants may be better reserved for seminar-style probing of “why” questions where richer reasoning—though not invariably more correct—is desirable. A pragmatic workflow would route simple nomenclature queries to the low-latency engines while invoking o3 or Gemini Pro for higher-order discussion prompts, thereby balancing speed with pedagogical rigour. To enhance translational usability, we provide a pragmatic selection matrix (Table 5) aligning classroom scenarios with model choices based on the measured accuracy-latency trade-offs. This matrix is intended as a teaching aid rather than a prescriptive standard.

**Table 5:** Pragmatic model selection for dental anatomy teaching.

Teaching scenario	Primary need	Recommended model	Reason	Cautions
Dissection labs, just-in-time look-ups	Speed	GPT-4o or Gemini Flash	Low latency; acceptable mid-tier accuracy	Use as a prompt, not an answer key, verify against atlas or lab tutor
Oral exam preparation, seminar “why” questions	Deeper reasoning	o3 (primary), Gemini Pro (alternate)	Highest overall accuracy	o3 is slow, use when pace allows
Self-study quizzes with feedback	Balance	Gemini Pro or GPT-4o	Moderate accuracy with manageable latency	Include human-written feedback and references
Musculoskeletal atlas drills	Max accuracy	o3	Top accuracy	Latency trade-off; schedule for guided sessions
Neurovascular drills	Fine structures	Gemini Pro or o3	Narrow top-tier accuracy	Consider region-of-interest prompting
High-stakes answer key	Reliability	None recommended	No model reached a safe threshold	Always require expert verification

Several limitations temper the generalisability of our findings. First, all images were taken from the anatomic atlas, a canonical but idealised source that omits pathological or population-specific variants. Expanding future benchmarks to include intra-operative photographs, endoscopic views, or cone-beam CT slices would broaden ecological validity. Second, the dataset comprises only 26 plates (260 labels), sufficient for overall statistics but underpowered for detailed subregion analyses, such as temporomandibular joints or paranasal sinuses, or tissue-specific strata, such as bones and vessels. Third, we evaluated models strictly in their default, unmodified configurations without any fine-tuning, system-prompt edits, tool augmentation, or hyperparameter changes. Additionally, unobserved vendor-side server load and provider-imposed request limits might have influenced latency and run-to-run consistency. Finally, our binary scoring scheme ignored partially correct answers. Adopting a graded rubric could capture incremental reasoning gains that are pedagogically meaningful even when the final nomenclature is imperfect. Future work should test larger, pathology-rich image sets from varied modalities and regions, benchmark retrieval-augmented or low-rank–adapted models, and apply a graded rubric that captures partial correctness while monitoring speed and stability.

## 5. Conclusions

Current publicly available multimodal LLMs recognise oral and maxillofacial landmarks with moderate accuracy at best. OpenAI o3 leads the cohort but sacrifices speed, while faster endpoints trade accuracy for latency. No model is yet reliable enough to serve as a stand-alone answer key, underscoring the need for domain-specific fine-tuning and continued human oversight. Future work should broaden image diversity, adopt graded scoring, and test hybrid vision–language pipelines to close this performance gap.

## Supporting information

S1 File Dataset. (XLSX)
